# Association Between Structural Housing Repairs for Low-Income Homeowners and Neighborhood Crime

**DOI:** 10.1001/jamanetworkopen.2021.17067

**Published:** 2021-07-21

**Authors:** Eugenia C. South, John MacDonald, Vincent Reina

**Affiliations:** 1 Urban Health Lab, Department of Emergency Medicine, Perelman School of Medicine at the University of Pennsylvania, Philadelphia; 2 Leonard Davis Institute, University of Pennsylvania, Philadelphia; 3 Department of Criminology, School of Arts and Sciences, University of Pennsylvania, Philadelphia; 4 Department of City and Regional Planning, Weitzman School of Design, University of Pennsylvania, Philadelphia

## Abstract

**Importance:**

The root causes of violent crime in Black urban neighborhoods are structural, including residential racial segregation and concentrated poverty. Previous work suggests that simple and scalable place-based environmental interventions can overcome the legacies of neighborhood disinvestment and have implications for health broadly and crime specifically.

**Objective:**

To assess whether structural repairs to the homes of low-income owners are associated with a reduction in nearby crime.

**Design, Setting, and Participants:**

This cross-sectional study using difference-in-differences analysis included data from the City of Philadelphia Basic Systems Repair Program (BSRP) from January 1, 2006, through April 30, 2013. The unit of analysis was block faces (single street segments between 2 consecutive intersecting streets) with or without homes that received the BSRP intervention. The blocks of homes that received BSRP services were compared with the blocks of eligible homes that were still on the waiting list. Data were analyzed from December 1, 2019, to February 28, 2021.

**Exposures:**

The BSRP intervention includes a grant of up to $20 000 provided to low-income owners for structural repairs to electrical, plumbing, heating, and roofing damage. Eligible homeowners must meet income guidelines, which are set by the US Department of Housing and Urban Development and vary yearly.

**Main Outcomes and Measures:**

The main outcome was police-reported crime across 7 major categories of violent and nonviolent crimes (homicide, assault, burglary, theft, robbery, disorderly conduct, and public drunkenness).

**Results:**

A total of 13 632 houses on 6732 block faces received the BSRP intervention. Owners of these homes had a mean (range) age of 56.5 (18-98) years, were predominantly Black (10 952 [78.6%]) or Latino (1658 [11.9%]) individuals, and had a mean monthly income of $993. These census tracts compared with those without BSRP intervention had a substantially larger Black population (49.5% vs 12.2%; |D| = 0.406) and higher unemployment rate (17.3% vs 9.3%; |D| = 0.357). The main regression analysis demonstrated that the addition to a block face of a property that received a BSRP intervention was associated with a 21.9% decrease in the expected count of total crime (incidence rate ratio [IRR], 0.78; 95% CI, 0.76-0.80; *P* < .001), 19.0% decrease in assault (IRR, 0.81; 95% CI, 0.79-0.84; *P* < .001), 22.6% decrease in robbery (IRR, 0.77; 95% CI, 0.75-0.80; *P* < .001), and 21.9% decrease in homicide (IRR, 0.78; 95% CI, 0.71-0.86; *P* < .001). When restricting the analysis to blocks with properties that had ever received a BSRP intervention, a total crime reduction of 25.4% was observed for each additional property (IRR, 0.75; 95% CI, 0.73-0.77; *P* < .001). A significant dose-dependent decrease in total crime was found such that the magnitude of association increased with higher numbers of homes participating in the BSRP on a block.

**Conclusions and Relevance:**

This study found that the BSRP intervention was associated with a modest but significant reduction in crime. These findings suggest that intentional and targeted financial investment in structural, scalable, and sustainable place-based interventions in neighborhoods that are still experiencing the lasting consequences of structural racism and segregation is a vital step toward achieving health equity.

## Key Points

**Question:**

Are targeted investments in structural repairs to homes of low-income owners associated with reduced crime in Black urban neighborhoods?

**Findings:**

In this cross-sectional study using difference-in-differences analysis of 13 632 houses on 6732 block faces in Philadelphia, Pennsylvania, the housing repair intervention analyzed was associated with a 21.9% reduction in total crime. Increasing the number of houses that received the intervention on a block was associated with a dose-dependent decrease in crime.

**Meaning:**

The results suggest that structural, scalable, and sustainable place-based interventions should be considered by policy makers who seek to address crime through non–police interventions.

## Teaser

This cross-sectional study examines the association between a housing renewal grant program implemented in socially disadvantaged, racially segregated communities in Philadelphia, Pennsylvania, and neighborhood crime.

## Introduction

In the US, violent crime is a salient public health problem that is largely concentrated in urban neighborhoods with predominantly Black residents.^1^ Homicide is the leading cause of death for Black men aged 1 to 44 years.^2^ Gun violence has become a resurgent issue in the country since the start of the COVID-19 pandemic; for example, in Philadelphia, Pennsylvania, the location of this study, shootings increased by 40% in 2020 from the year before.^3^ The health implications of violence exposure are vast and include increased risk of depression, posttraumatic stress disorder, and cardiovascular disease.^4,5,6,7,8,9^ People living in communities that experience spikes in violence have increased hospital visits and deaths from stress-responsive diseases.^10^ Pregnant women living in neighborhoods with high rates of violent crime report greater stress levels and have higher odds of preterm birth, which has lasting implications for the health and well-being of the child.^11,12^

The root causes of violent crime in Black urban neighborhoods are structural, including past and present racist policies and practices leading to residential racial segregation, concentrated poverty, and lack of economic opportunity.^13,14,15,16,17^ Neighborhoods in Philadelphia that were subjected to redlining in the first half of the twentieth century are the same neighborhoods with the highest concentration of violent crime today.^18^ One consequence of this cycle of concentrated racial disadvantage is the lack of neighborhood investment, which in turn is a factor in the deterioration of the neighborhood physical environment.^13,19^ A disinvested housing stock, blighted vacant lots, and a lack of greenspace are widespread environmental conditions that disproportionately occur in Black urban neighborhoods and are associated with stress, fear, poor mental health, and violence.^20,21,22,23^

Previous work has suggested that simple, scalable, and sustainable place-based environmental interventions can affect health broadly and crime specifically.^24,25^ For example, in a citywide randomized clinical trial, vacant lot trash cleanup and greening led to a reduction in gun assaults by more than 10% in neighborhoods with residents living below the poverty line.^26^ Tree canopy has been associated with a decrease in adolescent gun assault, and loss of tree canopy from invasive species has been associated with an increase in violent crime.^22,27^ A quasi-experimental analysis of remediating abandoned houses was associated with a substantial decrease in violent crime.^28^ These findings support the notion that the neighborhood physical environment shapes the social connectivity between neighbors, which in turn plays a role in preventing crime.^29,30^

Given the association between neighborhood structural interventions and crime reduction, we evaluated the association of structural repairs to owner-occupied homes with nearby crime. The City of Philadelphia Basic Systems Repair Program (BSRP) provides low-income homeowners with grants to repair structural damage to their homes.^31^ More than half of the housing units in Philadelphia were built before 1945, and aging houses are more likely to experience structural problems.^31^ However, for 36% of Philadelphia’s homeowners with annual household incomes less than $35 000, these problems linger and worsen over time because homeowners lack the resources to maintain or repair the damage to their homes. The BSRP was designed to address this problem. Such investment in the housing stock may be associated with other positive spillovers, including reduction in crime. We conducted a study of the BSRP to assess whether structural repairs to the homes of low-income owners are associated with a reduction in nearby crime. We hypothesized that this type of investment would be associated with decreased crime in the neighborhood.

## Methods

This cross-sectional panel time series used a difference-in-differences framework to analyze the City of Philadelphia BSRP data from January 1, 2006, through April 30, 2013. The institutional review boards of the University of Pennsylvania and City of Philadelphia approved the study. Informed consent was waived by the institutional review board of the University of Pennsylvania because administrative data were used and no direct contact with homeowners was made. We followed the Strengthening the Reporting of Observational Studies in Epidemiology (STROBE) reporting guideline.

### The BSRP Intervention

Operational since 1995, the BSRP is funded by the City of Philadelphia and run by the Philadelphia Housing Development Corporation, a nonprofit housing organization that works closely with the city to execute the program.^31^ The BSRP provides grants of up to $20 000 to low-income owners to fix structural emergencies in their owner-occupied homes, including electrical, plumbing, heating, and roofing damage. For example, structural repairs may include replacing exterior walls to stop leakage, and electrical repairs may include replacing circuits that overheat, spark, or do not stay on. A city contractor team completes the needed structural work.

To enroll in the BSRP, homeowners must apply and be screened for eligibility before being placed on a waiting list. The income guidelines are based on the Section 8 annual income limits set by the US Department of Housing and Urban Development and are revised annually.^32^ In 2020, for example, for a household size of 4 people, the annual income must be less than $48 300. The waiting time from enrollment to receipt of BSRP intervention has been 2 to 3 years. In this study, in addition to examining all blocks in Philadelphia, we compared the blocks of homes that had received BSRP services with the blocks of eligible homes that were still on the waiting list.

The City of Philadelphia provided the application data for the properties that participated in the BSRP during the study period. The address for each BSRP household was geocoded to the nearest property parcel. The parcel centroids were then joined to their associated block face (a single street segment between 2 consecutive intersecting streets) and census tracts.

### Outcome Measures

We used publicly available data from the Philadelphia Police Department to establish the outcome of police-reported crime. We created 7 major categories of crime (homicide, assault, burglary, theft, robbery, disorderly conduct, and public drunkenness), and we combined these categories to create a total crime variable. Crime categories included both violent and nonviolent crimes. The geocoded crime data were joined to their associated block face for all blocks in the city.

### Data Integration and Panel Data Set Creation

We used a k-nearest neighbor analysis to join the crime and BSRP data, using the get.knn function in R for values of latitude and longitude (R Foundation for Statistical Computing). Integrating these data produced a database that linked each crime to its nearest block face and that indicated whether that block face had a home that participated in the BSRP. Next, we aggregated the data for each block face for each quarter of the year to create a panel data set for the first quarter of 2006 (quarter 1) through the first quarter of 2013 (quarter 29).

Properties entered the panel data set when they were approved by the Philadelphia Housing Development Corporation to receive BSRP services and were designated as receiving the intervention after the structural work was completed. The period between entering the panel data set and receiving the intervention was the waiting list period. We started a cumulative running count of the number of houses on each block face that had received the BSRP intervention according to the quarter in which the intervention was provided. This panel data set allowed us to estimate the relative association of the concentration of BSRP services provided on each block face during the study period. Blocks on which a homeowner was wait-listed but ultimately received the BSRP intervention during the study period and blocks on which a homeowner was wait-listed but never received the intervention during the study period served as the control comparison for 1 of the analyses that we performed.

### Statistical Analysis

We described the demographic characteristics of the homeowners included in this analysis. We used the 2009 to 2013 five-year US Census Bureau American Community Survey to obtain neighborhood-level demographic information for the census tracts with homes that were served by the BSRP compared with the remainder of the city.

The unit of analysis was the block face. Block faces have long been recognized as a relevant unit of analysis for studies of crime and place given that social life is often organized around blocks.^33,34^ In addition, analyzing individual houses would introduce further measurement error because of houses clustering together. Given that the study outcome was police-reported crime, we used a difference-in-differences Poisson regression model to estimate the association of the BSRP with block-level crimes. The difference-in-differences approach reduces several threats to validity, including historical events and regression to the mean, and allows a better estimation of the true association of an intervention (BSRP) with an outcome (crime) in the absence of a prospectively designed trial. Poisson coefficients were converted to the incidence rate ratio (IRR) or the change in the ratio of incidents (counts) in response to the BSRP.

We included fixed effects for each block face to control for time-stable unmeasured differences between all blocks, which allowed us to identify the change attributed to the BSRP intervention. We also included quarterly and yearly fixed-effects regression estimates to control for yearly and seasonal patterns that are common over time across Philadelphia. The estimated mean change in crime per BSRP intervention was relative to the block faces with homes that were yet to receive or that never received the intervention, thus taking the form of a difference-in-differences design.^35^ We also analyzed a subset of blocks with homes that eventually received the intervention.

Hypothesis tests were 2-sided. The standardized mean difference, |D|, indicated the level of distance between 2 group means. Two-sided *P* < .05 indicated statistical significance.

All analyses were performed with Stata, version 15.1 (StataCorp LLC). Data were analyzed from December 1, 2019, to February 28, 2021.

## Results

We identified a total of 20 515 unique block faces nested within 1334 census block groups and 383 census tracts in Philadelphia during the study period. When multiplied by 29 quarters, the total increased to 594 935 unique block faces per quarter for the entire study panel. Approximately 646 block faces had 0 crime during the entire study period and were excluded from the analysis, leaving a total of 576 201 block faces for inclusion. Of the block faces in the city, 6732 of 19 869 (33.8%) had homes that had received the BSRP intervention during the study period.

A total of 13 632 houses received the intervention. The mean time on the BSRP waiting list was 2.58 years. The owners of these homes had a mean (range) age of 56.5 (18-98) years, were predominantly Black (10 952 [78.6%]) or Latino (1658 [11.9%]) individuals, and had a mean monthly income of $993.

**Figure 1.**
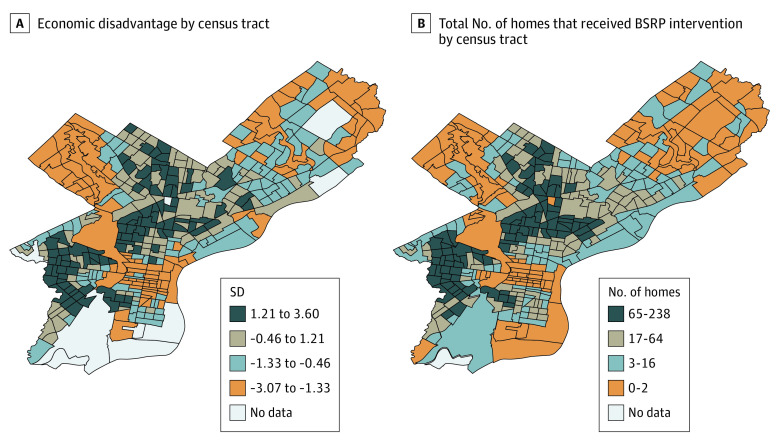
Distribution of Economic Disadvantage and Basic Systems Repair Program (BSRP) Intervention in Philadelphia Economic disadvantage by census tract is a combination of the percentages of Black residents, unemployed residents, homeowners with an annual income less than $25 000, and renters with an annual income less than $25 000 divided by quartile to show the least disadvantaged (orange) to most disadvantaged (dark blue) tract.

Figure 1 shows the distribution of homes that received the BSRP intervention in Philadelphia, demonstrating a clear clustering of services in lower-income Black neighborhoods. We evaluated the neighborhood-level sociodemographic characteristics of the census tracts that included at least 1 house with BSRP intervention (n = 307) (Table 1). In general, these neighborhoods compared with those without BSRP intervention had a substantially larger Black population (49.5% vs 12.2%; |D| = 0.406) and higher unemployment rate (17.3% vs 9.3%; |D| = 0.357), and they were located in tracts with a substantially higher percentage of homeowners with an income less than $25 000 per year (15.1% vs 4.8%; |D| = 0.501). Furthermore, these tracts had a Black home ownership rate that was more than 20 percentage points higher than the rate in tracts without BSRP intervention (25.1% vs 2.5%; |D| = 0.401).

**Table 1.** Neighborhood Demographic Differences Between Census Tracts With and Without Basic Systems Repair Program (BSRP) Intervention

Variable	With BSRP intervention		Without BSRP intervention		Standardized mean difference, D value
	No. of census tracts	Mean (SD), %	No. of census tracts	Mean (SD), %	
Resident race/ethnicity					
Black	307	49.5 (35.4)	70	12.2 (15.5)	0.406
White	307	30.8 (30.9)	70	69.3 (19.3)	0.458
Hispanic	307	12.4 (18.3)	70	5.5 (4.1)	0.157
Owner race/ethnicity					
Black	307	25.1 (22.0)	69	2.5 (4.1)	0.401
White	307	22.5 (23.7)	69	39.1 (25.2)	0.259
Hispanic	307	4.8 (8.7)	69	1.5 (2.1)	0.161
Unemployed rate	306	17.3 (8.3)	70	9.3 (8.0)	0.357
Owner annual income, $					
<25 000	307	15.1 (7.3)	69	4.8 (4.5)	0.501
25 000 to 50 000	307	13.4 (6.3)	69	8.4 (7.7)	0.282
>50 000 to 75 000	307	9.7 (5.1)	69	7.2 (6.1)	0.180
>75 000 to 100 000	307	6.4 (4.1)	69	6.5 (5.0)	0.007
>100 000	307	8.7 (8.1)	69	19.0 (13.6)	0.391

The main regression analysis showed that the addition to a block face of a property with BSRP intervention was associated with a significant reduction in total crime and all crime subcategories (Table 2). The addition to a block face of a property with BSRP intervention was associated with a 21.9% decrease in the expected count of total crime (IRR, 0.78; 95% CI, 0.76-0.80; *P* < .001), 19.0% decrease in assault (IRR, 0.81; 95% CI, 0.79-0.84; *P* < .001), 22.6% decrease in robbery (IRR, 0.77; 95% CI, 0.75-0.80; *P* < .001), and 21.9% decrease in homicide (IRR, 0.78; 95% CI, 0.71-0.86; *P* < .001). Although these estimates were large in magnitude, the mean count of these crimes per quarter was relatively low (0.884 per quarter). This count translated into roughly 5.6 fewer crimes in total over 29 quarters from the addition of a property with BSRP intervention.

**Table 2.** Association of Basic Systems Repair Program (BSRP) Intervention With Total Crime and Crime Subtypes by Block Face and by Blocks With Homes That Had Ever Received BSRP Intervention

Variable	Total	Burglary	Theft	Assault	Robbery	Homicide	Public drunkenness	Disorderly conduct
Block face								
Crime, IRR (95% CI)	0.78 (0.76-0.80)^a^	0.82 (0.80-0.85)^a^	0.75 (0.73-0.78)^a^	0.81 (0.79-0.84)^a^	0.77 (0.75-0.80)^a^	0.78 (0.71-0.86)^a^	0.70 (0.57-0.86)^a^	0.75 (0.70-0.81)^a^
Crime count/block, mean (SD)	0.884 (2.126)	0.148 (0.440)	0.521 (1.685)	0.169 (0.514)	0.170 (0.500)	0.041 (0.209)	0.071 (0.325)	0.150 (0.763)
Block face, No.	576 201	438 074	551 899	374 129	362 732	56 753	37 497	183 744
Block with homes that had ever received BSRP intervention								
Crime, IRR (95% CI)	0.75 (0.73-0.77)^a^	0.78 (0.76-0.81)^a^	0.73 (0.70-0.75)^a^	0.78 (0.75-0.80)^a^	0.72 (0.69-0.75)^a^	0.75 (0.67-0.83)^a^	0.66 (0.53-0.83)^a^	0.71 (0.67-0.76)^a^
Crime count/block, mean (SD)	0.863 (1.433)	0.166 (0.459)	0.401 (0.875)	0.190 (0.541)	0.150 (0.445)	0.040 (0.204)	0.038 (0.208)	0.097 (0.447)
Block face, No.	193 082	172 260	187 862	162 835	145 377	32 016	10 701	83 259

Abbreviation: IRR, incidence rate ratio.

^a^ *P* < .001.

**Figure 2.**
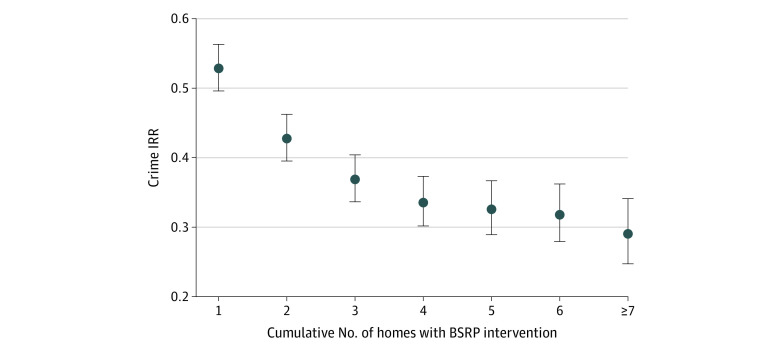
Dose-Dependent Association Between Basic Systems Repair Program (BSRP) Intervention and Crime Incidence rate ratio (IRR) represents the total expected count of crime for each additional home that received the BSRP intervention on a block face vs 0 homes. Circles indicate the IRRs; lines, 95% CIs.

We also restricted the analysis to blocks with homes that had ever received the BSRP intervention (Table 2). For this regression model, the difference-in-differences estimates were for the period after receipt of a BSRP grant compared with blocks with homes that were yet to receive the intervention. Consistent with the primary regression model, the results showed a reduction in the total expected count of crime of 25.4% (IRR, 0.75; 95% CI, 0.73-0.77; *P* < .001) for each additional property. We found a significant BSRP dose-dependent decrease in total crime such that the magnitude of impact increased with higher numbers of homes with BSRP intervention on a given block face (Figure 2). For example, addition of properties on a block face yielded an IRR for total crime of 0.53 for 1 home, 0.43 for 2 homes, 0.37 for 3 homes, and 0.34 for 4 homes.

We examined results by baseline crime quantiles (low, medium, or high) (Table 3). The association of the BSRP intervention with violent crime subtypes, such as robbery and homicide, appeared to be driven by the changes in areas in the highest crime quantile. To ascertain the robustness of the spatial clustering of BSRP block faces that were near each other, we assessed how much the SEs of the estimates would change for the primary models if regressions clustered the SEs at the census tract level.^36^ The results were unaffected by these adjustments (eMethods and eTable in the Supplement).

**Table 3.** Association of Basic Systems Repair Program (BSRP) Intervention With Crime Quantile

Variable	Total	Burglary	Theft	Assault	Robbery	Homicide	Public drunkenness	Disorderly conduct
Crime quantile: low								
Crime, IRR (95% CI)	0.88 (0.82-0.93)^a^	0.87 (0.79-0.97)^b^	0.86 (0.80-0.93)^a^	0.82 (0.72-0.92)^c^	1.12 (0.94-1.33)	0.81 (0.46-1.41)	1.31 (0.51-3.33)	0.99 (0.67-1.46)
Crime count/block, mean (SD)	0.141 (0.391)	0.058 (0.248)	0.093 (0.311)	0.051 (0.237)	0.044 (0.210)	0.037 (0.199)	0.036 (0.193)	0.039 (0.214)
Block face, No.	196 968	102 834	173 101	57 333	47 705	3654	2059	10 875
Crime quantile: medium								
Crime, IRR (95% CI)	0.87 (0.85-0.90)^a^	0.85 (0.81-0.89)^a^	0.85 (0.82-0.88)^a^	0.92 (0.87-0.96)^a^	0.88 (0.82-0.93)^a^	0.94 (0.78-1.14)	0.89 (0.55-1.45)	0.93 (0.82-1.06)
Crime count/block, mean (SD)	0.499 (0.763)	0.110 (0.354)	0.272 (0.551)	0.092 (0.339)	0.072 (0.272)	0.038 (0.198)	0.039 (0.210)	0.052 (0.258)
Block face, No.	184 237	158 224	183 802	133 574	127 310	13 050	5539	44 602
Crime quantile: high								
Crime, IRR (95% CI)	0.76 (0.73-0.78)^a^	0.81 (0.78-0.85)^a^	0.73 (0.70-0.75)^a^	0.79 (0.76-0.82)^a^	0.75 (0.72-0.78)^a^	0.74 (0.65-0.83)^a^	0.67 (0.53-0.84)^a^	0.73 (0.68-0.79)^a^
Crime count/block, mean (SD)	1.999 (3.273)	0.235 (0.563)	1.134 (2.659)	0.262 (0.649)	0.268 (0.633)	0.042 (0.213)	0.079 (0.348)	0.193 (0.894)
Block face, No.	194 996	177 016	194 996	183 222	187 717	40 049	29 899	128 267

Abbreviation: IRR, incidence rate ratio.

^a^ *P* < .001.

^b^ *P* < .05.

^c^ *P* < .01.

## Discussion

In this cross-sectional study using a difference-in-differences analysis, structural repairs to the homes of low-income owners were associated with a modest, but significant, reduction in crime at the block face level. The results included total crime and each crime category evaluated, including violent crime. We found a dose-dependent association between the concentration of homes that the BSRP served and a decrease in crime. Going stepwise from 1 to 4 participating homes on a block face showed a corresponding larger decrease in crime that leveled off from 5 to 7 homes. Given the design of this study and the regression model specification, the findings are suggestive of a potential causal association between this particular structural investment and crime in the neighborhood.

We believe these findings add much needed experimental evidence to the growing number of studies that call for structural, scalable, and sustainable interventions to address the legacy of racism and its lasting implications for health and safety.^24,37^ Environmental inequities, including vacant and blighted spaces, are directly associated with entrenched racial segregation, concentrated poverty, and economic disenfranchisement.^13,20^ Breaking the link between these long-standing forms of neighborhood disinvestment and the detrimental downstream implications for health will require an equally concentrated and sustained investment in Black neighborhoods. Environmental interventions, including vacant lot greening, abandoned house remediation, trash cleanup, and the BSRP, may represent evidence-based interventions that help break the association between structural racism and poor health.^26,28,38^ Investing in these interventions should be prioritized alongside other upstream structural solutions that address housing, education, health care, and criminal justice inequities.^37^

The BSRP is relatively low cost (each grant is less than $20 000), which should be an attractive feature to policy makers who make decisions on tight city budgets. Previous work has demonstrated the cost-benefit analysis of vacant lot greening and abandoned house remediation to reduce firearm violence.^39^ Future work should formally evaluate the cost savings from decreased crime associated with the BSRP.

There are several possible mechanisms involved in the association of structural home repairs with reductions in nearby crime, including the roles of social connectedness and stress, both of which are associated with crime.^40^ The spatial context of people’s living condition should be considered along with individual-based explanations for disease or poor health. Physical and social environments are associated with each other, and the behaviors and stress associated with these environments affect health.^41^ Previous qualitative work demonstrated that residents who lived in areas with high levels of physical deterioration were affected by their environment in several ways, including fracturing ties between neighbors and contributing to the experience of stigma.^21^ Vacant land remediation has been shown to increase socializing between neighbors, again demonstrating the link between physical environment and social connectedness. Collective efficacy, or the strength of relational community connections, has been associated with reduced crime^40^ and may increase on blocks where residents view the BSRP as an example of investment. Furthermore, structural repairs may provide stress relief for the owners and other occupants, including relief from financial stress associated with having limited means to pay for needed repairs and living in a home requiring considerable repairs. Lower levels of psychosocial stress may, in turn, mitigate or reduce disputes that otherwise could have led to acts of violence.^42^

The need for an intervention, such as the BSRP, is widespread in the US, in which more than 6 million houses are considered substandard by the Department of Housing and Urban Development.^43^ The Department of Housing and Urban Development defines a healthy home by the following 8 structural principles: dry, clean, safe, well ventilated, pest free, contaminant free, well maintained, and thermally controlled.^43,44^ In addition, affordable and high-quality housing has long been recognized as a key social determinant of health.^45^

Although not tested in this study, there may be direct health benefits associated with structural housing repairs. Deteriorating housing conditions are associated with a range of poor health outcomes, including injuries, respiratory disease, mental illness, and lead poisoning.^46,47,48,49,50,51^ Asthma, for example, is associated with the presence of mold and cockroaches, which are the result of excessive moisture from leaks, one of the repair options available through the BSRP.^49,51^ Depression is associated with poor exterior and interior housing quality.^50^ Further study of the BSRP is needed to evaluate potential direct health benefits.

Many activists and scholars have called for the shifting of funds away from local police departments, which are often among the top municipal budget items, and toward non–police interventions to respond to and prevent crime.^52,53^ Structural, scalable, and sustainable place-based interventions, including improving the quality of neighborhood housing through interventions, such as the BSRP, could be considered by policy makers who seek to address crime through non–police strategies.^24^ Targeted financial investment in neighborhoods that are still experiencing the lasting consequences of structural racism may be a vital step toward achieving health equity.

### Limitations

This study has several limitations. First, this study was conducted in Philadelphia, Pennsylvania, a large postindustrial, northeastern US city. The results may be most generalizable to other cities with a similar history of manufacturing job loss and resultant population decline, such as Baltimore, Maryland, or Detroit, Michigan. However, any city with low-income homeowners who lack access to home equity or other funds with which to make structural repairs may benefit from the study findings. Second, we did not evaluate the crime spillover to blocks beyond those served by the BSRP. However, past evaluation of place-based interventions found absolute reductions in crime rather than crime simply moving away from the site of intervention.^26^ Future studies that use local data are warranted. Third, although we accounted for time patterns using fixed effects, we may not have accounted for other spatial-temporal patterns that were associated with violent crime in a way that we could measure. We also likely undercounted violence by relying on police-reported crime given that certain domestic disputes or property crime may not be reported to the police.

## Conclusions

This study found that the BSRP intervention was associated with a modest, but significant, decrease in crime at the block face level. Policy makers who seek non–police interventions to respond to crime should consider structural, scalable, and sustainable place-based interventions, such as the BSRP. Financial investment in the physical environment of socioeconomically disadvantaged neighborhoods that are still experiencing the legacy of structural racism is a vital step toward achieving health equity.

## Notes

Supplement.
eMethods.
**eTable.** Sensitivity Estimates of Impact of BSRP Intervention on Total Crime and Crime Sub-Types by Block Face, Standard Errors Clustered at Block Group or Tract
eReference.
