# Supplementary material for: Association Between Structural Housing Repairs for Low-Income Homeowners and Neighborhood Crime
Source: JAMA Netw Open. 2021 Jul 21;4(7):e2117067. doi: 10.1001/jamanetworkopen.2021.17067 (PMC9435958; doi:10.1001/jamanetworkopen.2021.17067)
Supplement: Supplement. — eMethods. eTable. Sensitivity Estimates of Impact of BSRP Intervention on Total Crime and Crime Sub-Types by Block Face, Standard Errors Clustered at Block Group or Tract eReference. [file jamanetwopen-e2117067-s001.pdf]

Supplementary Online Content

South EC, MacDonald J, Reina V. Association between structural housing repairs for low-income homeowners and neighborhood crime. *JAMA Netw Open*. 2021;4(7):e2117067. doi:10.1001/jamanetworkopen.2021.17067

eMethods.

eTable. Sensitivity Estimates of Impact of BSRP Intervention on Total Crime and Crime Sub-Types by Block Face, Standard Errors Clustered at Block Group or Tract

eReference.

This supplementary material has been provided by the authors to give readers additional information about their work.

© 2021 South EC et al. *JAMA Network Open.*

eMethods.

For each regression model, we analyzed the relationship between the BSRP treatment on block face *i* in study quarter of the year *t* (Y_{it}). In each regression model, D is time varying interaction of BSRP counts and post treatment period. We include an indicator term for the block faces, α (*i*), where *i* denotes the block face (1,…19,218), which controls for omitted variables between blocks that are fixed in time. A total of 646 blocks are dropped from the analysis that have zero crime across all quarters. The regression models also control for seasonal trends by quarter and year using indicator terms, $\theta_{q(t)}$ and $\gamma_{y(t)}$, where q(t) denotes the quarter (1-4) y(t) the year (2006-2013). Ultimately, we estimate the effect of adding BSRP homes to block faces in the post-period relative to the pre-period compared to blocks that are yet to receive BSRP grants or never receive them. Each regression model is estimated as:

$$Y_{it} = \alpha_i + \theta_{q(t)} + \gamma_{y(t)} + \delta D_{i(t)} + \varepsilon_{it}$$

The regression models also control for dependence within the same block by clustering standard errors at the block face level (i).[1] We also examine how sensitive the results are to clustering standard errors at higher levels of geography represented by census block groups and census tracts. We also estimate the difference-in-differences regression models for quartiles of crime levels, the subset of blocks that ever receive BSRP grants, and for the presence of any BSRP grant.

We checked for parallel trends in crime prior to receiving the BSRP intervention among all blocks that eventually did receive the BSRP intervention. Using the quarter prior to BSRP implementation as the reference group, we used a chi-squared test of the 5 quarters leading up to implementation, and did not find a significant relationship between these prior quarters and the change in total crime (chi2 = 5.41, P=0.248) or any subcategories in crime. This implies that parallel trends are not a threat to the inference of the study.

© 2021 South EC et al. *JAMA Network Open.*

eTable. Sensitivity Estimates of Impact of BSRP Intervention on Total Crime and Crime Sub-Types by Block Face, Standard Errors Clustered at Block Group or Tract

	Total	Burglary	Theft	Assault	Robbery	Homicide	Drunk	Disorder
Crime	0.781***	0.823***	0.754***	0.810***	0.774***	0.781***	0.696***	0.754***
IRR [95% CI] Block group	[0.761, 0.801]	[0.798, 0.848]	[0.732, 0.776]	[0.785, 0.837]	[0.746, 0.803]	[0.708, 0.862]	[0.564, 0.858]	[0.705, 0.806]
IRR [95% CI] Tract	[0.761, 0.801]	[0.798, 0.848]	[0.732, 0.776]	[0.785, 0.837]	[0.746, 0.803]	[0.708, 0.861]	[0.565, 0.858]	[0.705, 0.805]
Crime count/ block, mean	0.884	0.148	0.521	0.169	0.170	0.0407	0.0710	0.150
Block face, n	576201	438074	551899	374129	362732	56753	37497	183744

IRR = Incident rate ratios
CI = confidence interval
n = number
*** $p < 0.001$

© 2021 South EC et al. *JAMA Network Open.*

eReference.

1. Bester CA, Conley TG, Hansen CB. Inference with dependent data using cluster covariance estimators. *J Econom*. 2011. doi:10.1016/j.jeconom.2011.01.007

© 2021 South EC et al. *JAMA Network Open.*
